# Pharmacological Inhibition of p38 MAPK by SB203580 Increases Resistance to Carboplatin in A2780cp Cells and Promotes Growth in Primary Ovarian Cancer Cells

**DOI:** 10.3390/ijms19082184

**Published:** 2018-07-26

**Authors:** Xiaolu Han, Huachen Chen, Jiesi Zhou, Helen Steed, Lynne-Marie Postovit, YangXin Fu

**Affiliations:** 1 Department of Oncology, Faculty of Medicine and Dentistry, University of Alberta, Edmonton, AB T6G 2E1 Canada; xiaolu8@ualberta.ca (X.H.); huachen2@ualberta.ca (H.C.); jiesi@ualberta.ca (J.Z.); postovit@ualberta.ca (L.-M.P.); 2 Department of Obstetrics and Gynecology, Faculty of Medicine and Dentistry, University of Alberta, Edmonton, AB T6G 2E1, Canada; Helen.Steed@albertahealthservices.ca

**Keywords:** epithelial ovarian cancer, chemoresistance, carboplatin, p38 MAPK, primary EOC cells, surviving

## Abstract

Chemoresistance renders current chemotherapy regimens ineffective against advanced epithelial ovarian cancer (EOC). Carboplatin (the first-line chemotherapeutic agent to treat EOC) induces cell death by regulating multiple signaling pathways. The objective of this study is to identify the signaling pathways that contribute to carboplatin resistance in EOC. To this end, we performed a proteome profiler human phospho-kinase array experiment and compared the phosphorylation profiles between the cisplatin-sensitive A2780s versus its derivative cisplatin-resistant A2780cp cells. The phospho-kinase array revealed that A2780s and A2780cp cells displayed different profiles in basal and carboplatin-induced phosphorylation. Phosphorylation of p38 MAPK was increased by carboplatin more markedly in A2780s cells compared to A2780cp cells. Inhibition of p38 MAPK activity by its specific inhibitor SB203580 increased resistance to carboplatin in A2780cp cells, but not in A2780s cells or in ascites-derived high-grade serous EOC cells. Interestingly, SB203580 increased the number of viable cells in the primary EOC cells, which was concomitant with an increase in survivin expression. In conclusion, inhibition of p38 MAPK by SB203580 increases resistance to carboplatin in A2780cp cells and the number of viable cells in the primary EOC cells, suggesting that pharmacological inhibition of p38 MAPK might not be an effective therapeutic strategy for EOC.

## 1. Introduction

Epithelial ovarian cancer (EOC) comprises approximately 90% of ovarian cancer, the most lethal gynecologic malignancy worldwide [1]. High-grade serous EOC is the most common and lethal histological subtype, accounting for approximately 75% of all EOC cases [2]. Most EOC cases are diagnosed at advanced stages and require a combination of surgery and chemotherapy (carboplatin in combination with paclitaxel being the first-line treatment). However, despite the initial positive response to chemotherapy, most EOCs relapse and develop chemoresistance, rendering current therapeutic regimens ineffective against advanced EOC. As a result, the current 5-year survival rate for advanced EOC is about 30% [3]. There is a need to better understand the molecular mechanisms of carboplatin resistance in order to develop novel therapeutic strategies and improve the management of EOC. 

Platinum-based compounds, including cisplatin and carboplatin, have been the first-line chemotherapeutic agents to treat EOC for several decades [4]. Carboplatin, the second generation of platinum drug, is currently more often used in clinic due to its low toxicity profile when compared to cisplatin [5]. Cisplatin and carboplatin share similar modes of action and mechanisms of resistance and induce cytotoxicity through multiple mechanisms [6,7,8,9]. Upon activation in the cell, the reactive cisplatin covalently binds to DNA and forms inter- and intra-strand adducts that will activate the DNA damage response and the DNA repair pathways. If the damage is beyond the repair capacity, cell death pathways will be activated [6,7,8,9]. DNA damage-induced cell death signaling cascades includes activation of ATM (ataxia telangiectasia mutated protein), ATR (ataxia telangiectasia and Rad3-related protein) and CHEK1 (checkpoint kinase 1), as well as their downstream signaling molecules, such as p53 and MAP kinases [6,7,8,9]. Cancer cells develop platinum resistance through multiple mechanisms, including enhanced DNA damage repair and activation of survival signaling pathways [6,7,8,9]. Similarly, as reviewed recently by Cornelison et al., multiple mechanisms are involved in chemoresistance in EOC, highlighting the need to develop therapeutic strategies that target more than one mechanism [10]. Platinum activates multiple signaling pathways and the balance between the death and survival signaling pathways determines the fate of the cells [6,7,8,9]. Activation of p38 mitogen-activated protein kinase (MAPK), which regulates an array of downstream targets, plays a dual role in cancer: it can either promote or suppress growth, metastasis, and chemoresistance in a context-dependent manner [11,12,13]. Indeed, platinum-induced p38 MAPK activation has been demonstrated to be either pro-apoptotic or pro-survival in various types of cancer [14]. Targeting p38 has been proposed as a potential therapeutic approach to treat several types of cancer [11,12]. In terms of platinum resistance in EOC, most studies used established EOC cell lines and determined that activation of p38 MAPK is pro-apoptotic in cisplatin-induced cytotoxicity in EOC cells [15,16,17,18,19]. Additionally, activation of p38 MAPK has been shown to be involved in apoptosis induced by several other antitumor agents in cisplatin-resistant EOC cells [20,21,22,23,24]. On the other hand, one recent study showed that activation of p38 MAPK contributes cisplatin resistance in EOC cells [25]. However, the functional implication of p38 MAPK activation has not been studied using primary EOC cells yet. 

In this study, using a proteome profiler human phospho-kinase array, we showed that cisplatin-sensitive A2780s and its derivative cisplatin-resistant A2780cp cells displayed different basal and carboplatin-induced phosphorylation profiles in proteins involving multiple signaling pathways. Among the differentially phosphorylated proteins, p38 MAPK phosphorylation was more markedly increased by carboplatin in A2780s cells compared to A2780cp cells. Using the specific p38 MAPK inhibitor SB203580, we demonstrated that inhibition of p38 MAPK increased resistance to carboplatin in A2780cp cells, but not in A2780s cells. Because the A2780 cell line has been shown to be a poor model for high-grade serous EOC [26] and high-grade serous EOC is the most common and lethal histological subtype [2], we examined the effect of SB203580 in primary high-grade serous EOC cells isolated from patient’s ascites. We determined that p38 MAPK phosphorylation was induced by carboplatin in primary high-grade serous EOC cells that displayed a variable sensitivity to carboplatin. Inhibition of p38 MAPK by SB203580 had minimal or no effect on carboplatin-induced cytotoxicity in primary EOC cells. However, interestingly, SB203580 treatment increased the number of viable cells in the primary EOC cells, which was concomitant with increased survivin expression. This is the first study to examine the effect of p38 MAPK on growth and carboplatin sensitivity in primary EOC cells. 

## 2. Results 

### 2.1. Different Phosphorylation Profiles between A2780s and A2780cp Cells

Our previous study determined that cisplatin-resistant A2780cp cells are also more resistant to carboplatin when compared to A2780s cells [27]. Because carboplatin is currently more often used in clinic compared to cisplatin [5], we used carboplatin for this study. To determine the phosphorylation profiles in A2780s and A2780cp cells upon carboplatin treatment, we left the cells untreated or treated them with 50 µM carboplatin for 24 h and used the cell lysates for a phospho-kinase array analysis. The array results showed that A2780s and A2780cp cells displayed different phosphorylation profiles at the basal level and after carboplatin treatment (Supplementary Figures S1 and S2). The phosphorylated proteins were quantified and divided into two groups. In the first group, protein phosphorylation was induced by carboplatin in A2780s cells, but it was less pronounced in A2780cp cells (Supplementary Figure S2A). In the second group, the basal level of protein phosphorylation was different between A2780s and A2780cp cells, but it was not affected by carboplatin treatment (Supplementary Figure S2B). 

**Figure 1:**
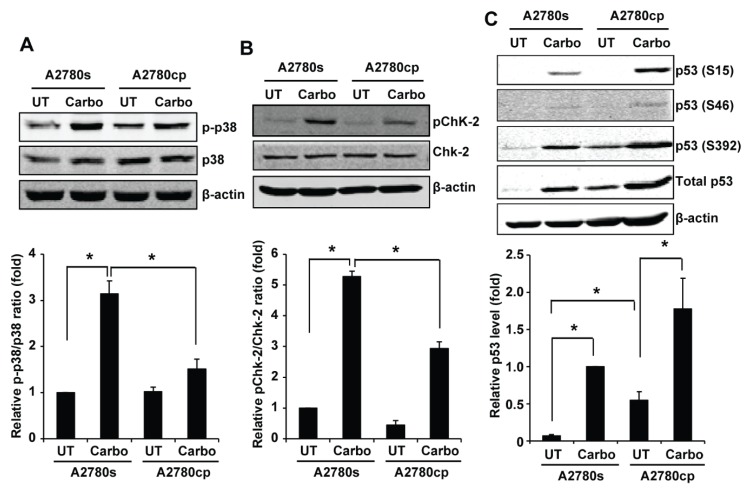
Validation of the kinase array data by Western blotting. A2780s and A2780cp cells were left untreated or treated with 50 μM carboplatin for 24 h, and the whole cell lysates were collected for Western blotting to measure total and phosphorylated p38 (**A**), Chk-2 (**B**), and p53 (**C**). β-actin was used as the loading control. (**A**,**B**) Phosphorylated p38 (p-p38) and Chk-2 (pChk-2) were quantified using Odyssey imaging software and normalized against total p38 and Chk-2. Relative p-p38/total p38 and pChk-2/total Chk-2 ratio (fold change) were shown as mean ± SE of four and three independent experiments, respectively, with that in untreated A2780s cells designated as 1. (**C**) Total p53 was quantified using Odyssey imaging software and normalized against β-actin and expressed as relative fold change with that in carboplatin-treated A2780s cells designated as 1. The relative p53 level was shown as mean ± SE of three independent experiments. * Significantly different (*p* < 0.05).

We selected the proteins in the first group for validation by Western blotting. We left A2780s and A2780cp cells untreated or treated them with 50 µM carboplatin for 24 h, and used the whole cell lysates for Western blotting. p38 MAPK regulates an array of downstream targets and activation of p38 MAPK can either promote or suppress growth, metastasis, and chemoresistance in a context-dependent manner [11,12,13]. Consistent with the kinase array results, Western blotting showed that p38 MAPK was phosphorylated in both A2780s and A2780cp cells; however, its phosphorylation was more markedly increased by carboplatin in A2780s cells compared to A2780cp cells (Figure 1A). Quantification of p38 MAPK phosphorylation showed that carboplatin-induced p38 MAPK phosphorylation in A2780s was two-fold higher compared to A2780cp cells (Figure 1A). 

Checkpoint kinase 2 (Chk2) is activated by ATM via phosphorylation at Thr-68 and mediates cisplatin-induced cell death [28]. In keeping with the kinase array results, our Western blotting showed that carboplatin induced Chk2 phosphorylation at threonine 68 (T68) in both A2780s and A2780cp cells; however, the induction was more pronounced in A2780s cells compared to A2780cp cells (Figure 1B). We also validated p53 phosphorylation by Western blotting. p53 is known to be activated by cisplatin [6,7,8,9]. Western blotting confirmed that carboplatin induced phosphorylation of p53 at multiple serine sites (S15, S46 and S392) in both A2780s and A2780cp cells and the basal level of p53 phosphorylation was more pronounced in A2780cp cells compared to A2780s cells. Western blotting showed that the basal level of p53 protein was higher in A2780cp cells compared to A2780s cells, and carboplatin significantly increased p53 protein levels in both A2780s and A2780cp cells (Figure 1C). These data suggest that more pronounced p53 phosphorylation observed in A2780cp cells was not due to increased phosphorylation per se, but rather due to an increase in p53 protein level. 

### 2.2. Inhibition of p38 MAPK Decreases Carboplatin-Induced Cytotoxicity in A2780cp Cells

**Figure 2:**
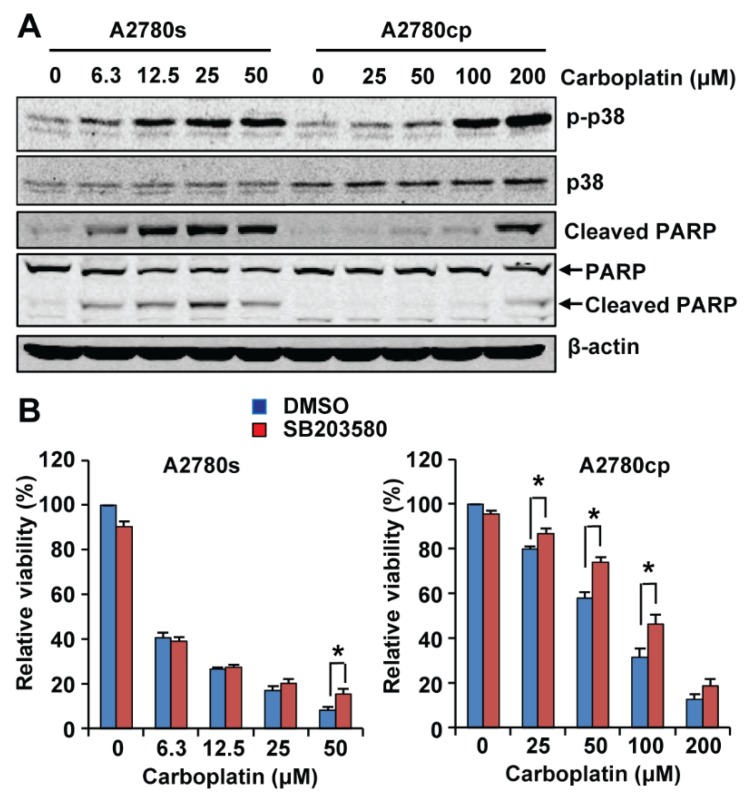
Effect of p38 MAPK inhibition by SB203580 on carboplatin sensitivity in A2780s and A2780cp cells. (**A**) A2780s and A2780cp cells were treated with increasing concentrations of carboplatin for 48 h. Phosphorylation of p38 and cleavage of Poly(ADP-ribose) polymerase (PARP) were analyzed by Western blotting. Two antibodies were used to examine the cleaved PARP: an antibody that only recognizes the cleaved PARP (**top panel**) and an antibody that recognizes both full-length and cleaved PARP (the **lower panel**). Both antibodies showed the same results. β-actin was used as the loading control. Two independent experiments showed the same results. (**B**) A2780s and A2780cp cells were treated with increasing concentrations of carboplatin in the presence of SB203580 (10 μM) or an equal volume of DMSO (the vehicle control) for 72 h. Cell viability was determined by the neutral red uptake assay. Data are shown as mean ± SE of seven independent experiments. * Significantly different (*p* < 0.05).

We selected p38 MAPK for further analysis because the functional impact of p38 MAPK activation on cisplatin resistance in EOC remains controversial [15,16,17,18,19,25] and has not been studied using primary EOC cells. To determine the effect p38 MAPK phosphorylation on carboplatin-induced cytotoxicity, we first treated A2780s and A2780cp cells with increasing concentrations of carboplatin for 48 h and determined phosphorylation of p38 MAPK and cleavage of PARP (Poly(ADP-ribose) polymerase), a marker for apoptosis, by Western blotting. As shown in Figure 2A, carboplatin induced phosphorylation of p38 MAPK in a dose-dependent manner in both A2780s and A2780cp cells; however, a higher dose of carboplatin was required to induce p38 MAPK phosphorylation in A2780cp cells (Figure 2A). PARP cleavage was induced by carboplatin at as low as 6.3 µM in A2780s cells, but was observed in A2780cp cells only when they were treated with 200 µM carboplatin (Figure 2A), which is consistent with our previous observation that A2780cp cells are more resistant to carboplatin-induced cytotoxicity than A2780s cells [27]. 

We then treated A2780s and A2780cp cells with increasing concentrations of carboplatin in the presence of 10 µM SB203580 (a specific p38 MAPK inhibitor) or an equal volume of Dimethyl Sulfoxide (DMSO) (the vehicle control) for 72 h and determined the cell viability using the neutral red uptake assay as we previously described [27]. Our results showed that inhibition of p38 MAPK by SB203580 did not change the overall sensitivity of A2780s cells to carboplatin-induced cytotoxicity (Figure 2B). SB203580 increased the viability of A2780s cells only when they were treated with the highest dose (50 µM). However, SB203580 co-treatment rendered A2780cp cells more resistant to carboplatin cytotoxicity (Figure 2B), increasing the IC_50_ for carboplatin from 60.6 to 89.0 µM in A2780cp cells. Our results suggest that p38 MAPK activation is dispensable for carboplatin-induced cytotoxicity in A2780s cells, but it is partially required in A2780cp cells. 

### 2.3. Inhibition of p38 MAPK Increases the Number of Viable Cells in Primary EOC Cells

**Figure 3:**
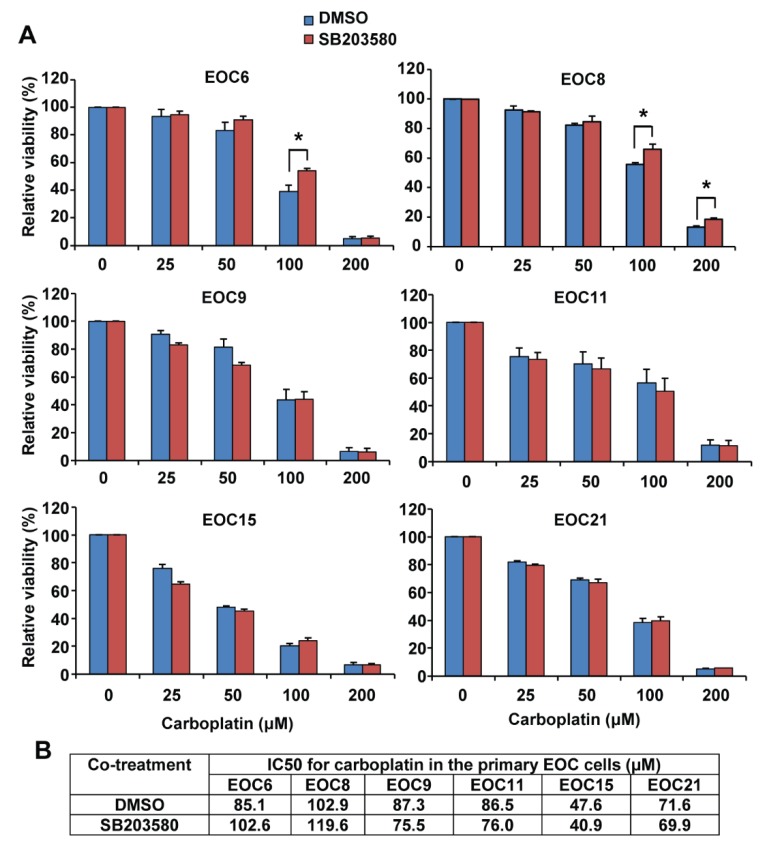
Inhibition of p38 MAPK by SB203580 has minimal or no effect on carboplatin sensitivity in primary epithelial ovarian cancer (EOC) cells. Primary EOC cells isolated from ascites of six patients with high-grade serous EOC were cultured in adherent condition for one day and then treated with increasing concentrations of carboplatin in the presence of SB203580 (10 μM) or an equal volume of DMSO (the vehicle control) for 72 h. Cell viability was determined by the neutral red uptake assay. (**A**) Data are shown as mean ± SE of three to five independent experiments. * Significantly different (*p* < 0.05). (**B**) The IC_50_ for carboplatin in these primary EOC cells in the presence of SB203580 or DMSO is shown.

**Figure 4:**
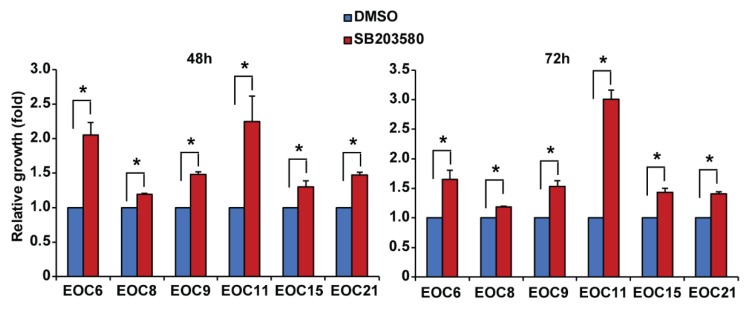
Inhibition of p38 MAPK by SB203580 increases the number of viable cells in primary EOC cells. Primary EOC cells were cultured in adherent condition and treated with 10 μM SB203580 or an equal volume of DMSO (the vehicle control). Viable cells were measured at 48 and 72 h using the neutral red uptake assay and expressed as fold changes (SB203580 versus DMSO). Data are shown as mean ± SE of three independent experiments. * Significantly different (*p* < 0.05).

High-grade serous EOC is the most common and lethal histological subtype [2]. However, the A2780 cell line has been shown to be a poor model for high-grade serous EOC [26]. We therefore used six primary high-grade serous EOC cells isolated from patients’ ascites in this study. These cells display different morphology, indicating the heterogeneity in cell morphology among the samples (Supplementary Figure S3). We confirmed that the ascites-derived cells express paired-box gene 8 (PAX8, a marker for serous ovarian cancer) as determined by immunocytochemistry and Western blotting (Supplementary Figures S4 and S5) [29,30,31], as well as cytokeratins as determined by Western blotting using a pan-keratin antibody (Supplementary Figure S5) as we previously reported in EOC cell line SKOV3 and OVCA429 cells [32]. These data thus confirmed the EOC origin of the ascites-derived cells. We found that the primary EOC cells obtained from patients before treatment (EOC6 and EOC9) (Table 1) grew faster than the cells obtained from the recurrent patients who had received treatment (EOC8, EOC11, EOC15, and EOC21) (Table 1). Specifically, after three days in culture, the viable cell numbers as determined by the neutral red uptake assay were increased by 2.2 and 2.7 fold in EOC6 and EOC9, respectively, and by 2.0, 1.8, 1.4, and 1.5 fold in EOC8, EOC11, EOC15, and EOC21, respectively. Interestingly, EOC6 and EOC9 displayed a cobblestone morphology, whereas EOC8, EOC15, and EOC21 displayed a more spindle morphology (Supplementary Figure S3). The primary EOC cells were treated with increasing concentrations of carboplatin in the presence of 10 µM SB203580 or an equal volume of DMSO for 72 h. As shown in Figure 3, these primary cells displayed variable sensitivity to carboplatin. Regardless of the sensitivity to carboplatin, SB203580 co-treatment did not significantly change the response of the primary EOC cells to carboplatin (Figure 3A,B). Specifically, SB203580 slightly increased the IC_50_ for carboplatin in EOC6 and EOC8, but it did not change the IC_50_ in the other four primary EOC cells. However, interestingly, we found that treatment of SB203580 at 10 µM for 48 or 72 h increased the number of viable cells in all primary EOC cells (Figure 4). 

### 2.4. Inhibition of p38 MAPK Increases Survivin Expression in Primary EOC Cells

**Figure 5:**
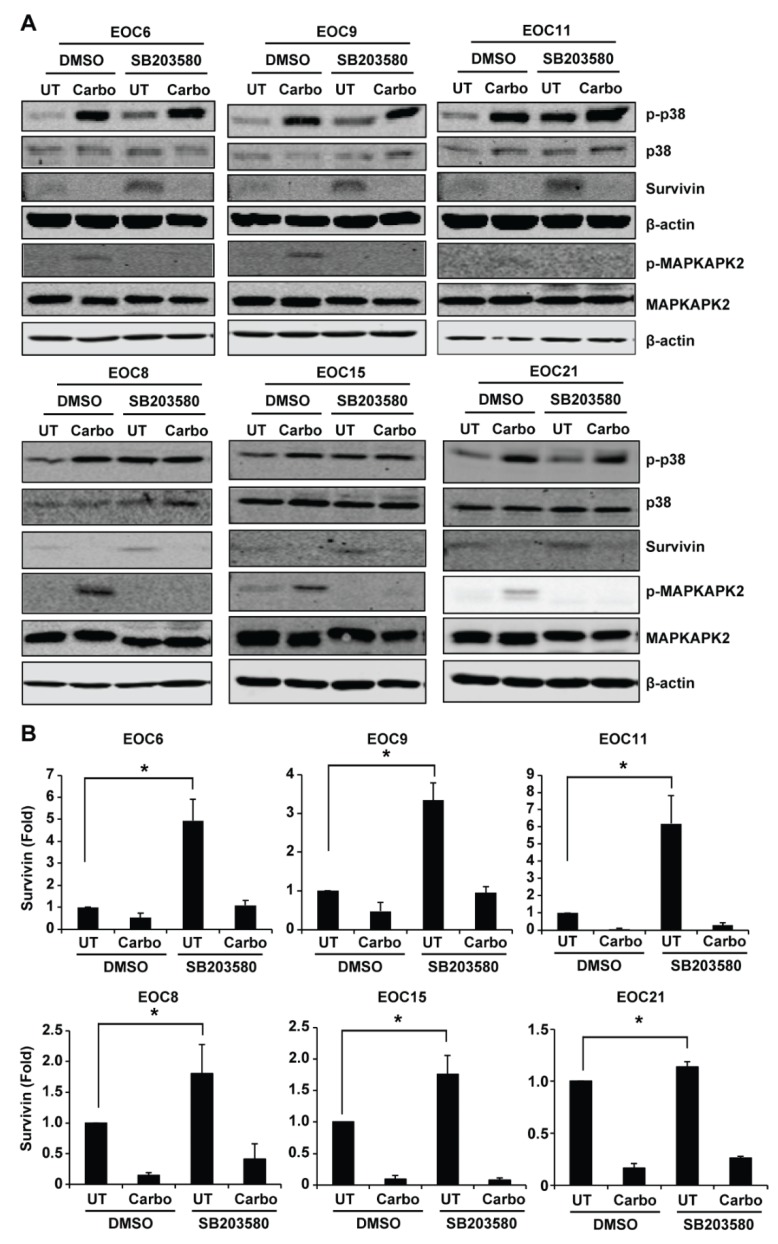
Inhibition of p38 MAPK by SB203580 increases survivin expression in primary EOC cells. Primary EOC cells were left untreated or treated with 200 μM carboplatin in the presence of SB203580 (10 μM) or an equal volume of DMSO (the vehicle control) for 48 h. (**A**) Phosphorylation of p38 MAPK and MAPKAPK2 (a direct substrate target of p38 MAPK), as well as survivin were examined by Western blotting. β-actin was used as the loading control. (**B**) The Western blotting results of survivin were quantified using Odyssey imaging software. The density of the survivin bands was normalized to that of β-actin. The density of the bands in the DMSO/UT cells was designated as 1. The relative level (fold change) of survivin was shown as mean ± SE of three to six independent experiments. * Significantly different (*p* < 0.05).

Western blotting confirmed that carboplatin induced p38 MAPK phosphorylation in all primary EOC cells (Figure 5). Survivin is implicated in cisplatin-induced cytotoxicity in EOC [33,34]. Because published studies showed that p38 MAPK mediates survivin downregulation and apoptosis induced by tanshinone IIA and a high dose of nitric oxide in EOC cells [21,35,36], we examined how carboplatin and SB203580 treatment affects survivin expression in the primary EOC cells. Western blotting showed that SB203580 increased the expression of survivin in all primary EOC cells compared to the DMSO control, albeit to a variable extent (Figure 5). Quantification of the survivin bands of three to six independent experiments showed that SB203580 treatment increased survivin protein level by 4.93 fold in EOC6, 1.80 fold in EOC8, 3.35 fold in EOC9, 6.18 fold in EOC11, 1.76 fold in EOC15, and 1.14 fold in EOC21 when compared to DMSO treatment. Carboplatin decreased survivin expression in these cells, and SB203580 treatment was insufficient to overcome this downregulation (Figure 5). SB203580 is a specific p38 MAPK inhibitor that inhibits its catalytic activity, but not its phosphorylation and activation by upstream kinases [37]. To confirm that increase of survivin by SB203580 is due to inhibition of p38 MAPK activity, we measured phosphorylation of MAPKAPK2 (a direct target substrate of p38 MAPK). Indeed, carboplatin-induced MAPKAPK2 phosphorylation was abolished by SB203580 in the primary EOC cells (Figure 5), indicating that SB203580 effectively inhibited p38 MAPK activity (Figure 5). Our results suggest that activation of p38 MAPK negatively regulates survivin expression in primary EOC cells. 

### 2.5. Inhibition of p38 MAPK Increases the Number of Viable Cells in Primary EOC Cells Cultured in Spheroids

**Figure 6:**
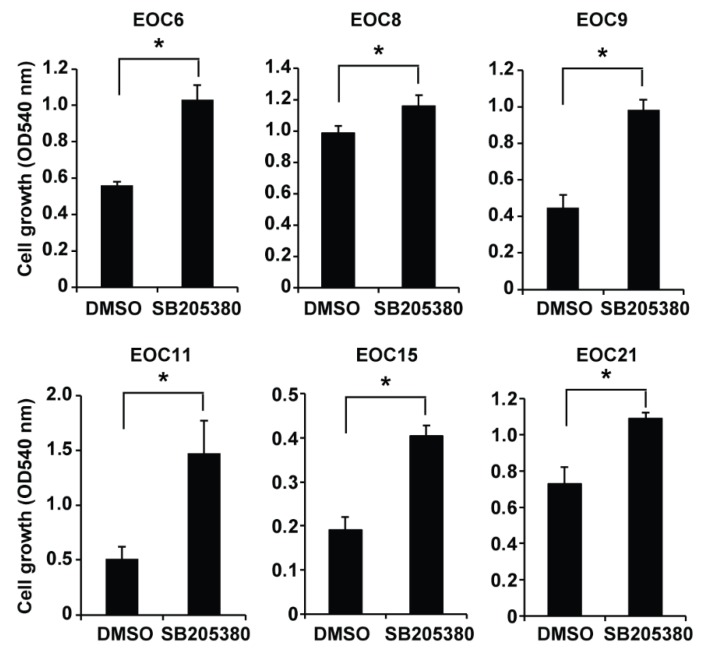
Inhibition of p38 MAPK by SB203580 increases the number of viable cells in the primary EOC cells cultured in spheroids. Primary EOC cells were cultured in spheroids in 24-well low attachment plates (50,000 cells/well) and treated with 10 μM SB203580 or an equal volume of DMSO (the vehicle control) for three days. The number of viable cells was determined using the neutral red uptake assay and shown as absorbance at 540 nm. Data are shown as mean ± SD of three spheroid cultures. * Significantly different (*p* < 0.05).

EOC cells disseminate from the original tumors and survive as spheroid in ascites before they colonize on peritoneal tissues [38]. To determine whether p38 MAPK activation affects the growth of the primary EOC cells cultured in spheroids, we cultured primary EOC cells in ultra-low attachment plates to allow them to form spheroids in the presence of 10 µM SB203580 or an equal volume of DMSO for three days. The primary EOC cells formed spheroids as shown at the 24 h timepoint, and the spheroids aggregated to form larger and more compact clusters afterwards as shown at the 48 and 72 h timepoints (Supplementary Figure S6), which prohibited an accurate measurement of the number and size of the spheroids. We therefore used the neutral red uptake assay to measure the viable cells in the spheroids/clusters. Consistent with the cells in adherent culture, SB203580 treatment increased the number of viable cells in all primary EOC cells cultured in spheroids compared to the DMSO control (Figure 6). Our results indicate that inhibition of p38 MAPK increases the number of viable cells in the primary EOC cells in both adherent culture and in spheroids. 

## 3. Discussion 

Cisplatin induces DNA damage, which results in the activation of multiple signaling pathways via phosphorylation of critical signaling proteins [6,7,8,9]. In this study, using a phospho-kinase array, we demonstrated that cisplatin-sensitive A2780s and cisplatin-resistant A2780cp cells display different profiles in basal and carboplatin-induced phosphorylation of certain signaling proteins (Supplementary Figures S1 and S2), indicating that signaling pathways are differentially activated in these two cell lines. We chose the proteins whose phosphorylation was induced by carboplatin (Supplementary Figure S2A) for validation using Western blotting. Chk2 is activated by ATM via phosphorylation at Thr-68 and mediates cisplatin-induced cell death [28]. In keeping with the kinase array data, our Western blotting confirmed that induction of Chk2 phosphorylation by carboplatin is more pronounced in carboplatin-sensitive A2780s cells compared to carboplatin-resistant A2780cp cells (Figure 1B). This finding is consistent with a recent report that high expression of Chk2 is associated with better response towards platinum-based chemotherapy in EOC patients, and knockdown of Chk2 renders ovarian cancer more resistant against platinum-based chemotherapy [39]. Therefore, impaired ChK2 phosphorylation in A2780cp can be a mechanism by which A2780cp cells becomes resistance to cisplatin-induced cell death. We also validated p53 phosphorylation by carboplatin in A2780s and A2780cp cells. p53 is known to be activated by cisplatin [6,7,8,9]. Western blotting showed that the basal level of p53 protein is higher in A2780cp cells compared to A2780s cells, and it is further increased in both cell types upon carboplatin treatment. In keeping with p53 protein levels, p53 phosphorylation at S15, S46, and S392 is increased by carboplatin in both cell types; however, the phosphorylation is more pronounced in A2780cp cells compared to A2780s cells. Because the A2780cp cell line used in this study has a mutated p53 (V172F and R260S), carboplatin-induced p53 phosphorylation may not necessarily lead to activation of the downstream signaling events in A2780cp cells [40]. 

Our Western blotting confirmed that p38 MAPK is phosphorylated in both A2780s and A2780cp cells and its phosphorylation is more markedly increased by carboplatin in A2780s cells compared to A2780cp cells. Thus far, studies carried out in established EOC cell lines have reported contradictory results regarding the role of p38 MAPK in cisplatin resistance in EOC. While most studies showed that activation of p38 MAPK contributes to cytotoxicity in EOC cells [15,16,17,18,19,20,21,22,23,24], one recent study reported that inhibition of p38 MAPK sensitizes EOC cells to cisplatin [25]. In this study, we found that activation of p38 MAPK is dispensable for carboplatin-induced cell death in cisplatin-sensitive A2780s cells, but it is partially required for carboplatin-induced cell death in cisplatin-resistant A2780cp cells. Carboplatin induces cell death via multiple mechanisms [6,7,8,9]. We speculate that in A2780s cells, activation of other pro-apoptotic signaling pathways by carboplatin renders p38 MAPK activation dispensable for carboplatin-induced cell death. However, in A2780cp cells, where other pro-apoptotic signaling pathways are altered or compromised, activation of p38 MAPK becomes a relevant event in carboplatin-induced cell death. 

High-grade serous EOC is the most common and aggressive histological subtype of EOC [2]. Thus far, the role of p38 MAPK in carboplatin sensitivity has not been examined in primary EOC cells. To address this paucity, we examined the effect of pharmacological inhibition of p38 MAPK on carboplatin sensitivity in primary EOC cells isolated from ascites of high-grade serous EOC patients. These ascites-derived cells are PAX8 positive and express cytokeratins (Supplementary Figures S4 and S5), confirming that they are EOC cells. Our results showed that primary EOC cells display a broad range of sensitivity to carboplatin. We have previously determined that the IC_50_ for carboplatin is 13.6 µM in cisplatin-sensitive A2780s cells and 150.8 µM in cisplatin-resistant A2780cp cells using the neutral red uptake assay [27]. In this study, we determined that the IC_50_ for carboplatin ranges from 47.6 to 102.9 µM in six ascites-derived primary EOC cells, which falls between A2780s and A2780cp cells. Despite the variable sensitivity to carboplatin, p38 phosphorylation is similarly induced by carboplatin in all the primary EOC cells. Inhibition of p38 MAPK by SB203580, however, has minimal or no effect on their response to carboplatin treatment, suggesting that p38 MAPK activation is dispensable for carboplatin-induced cell death in these primary EOC cells. SB203580 inhibits the catalytic activity, but not phosphorylation of p38 MAPK [37]. We confirmed that SB203580 effectively inhibits p38 MAPK activity in the primary EOC cells in this study because SB203580 abolished phosphorylation of MAPKAPK2 (a direct substrate target of p38 MAPK) in the primary EOC cells. Although we have only a small sample size to draw a conclusion, we did observe the following. First, cells obtained from patients before treatment (EOC6 and EOC9) grow faster than the cells obtained from the recurrent patients who had received treatment (EOC8, EOC11, EOC15, and EOC21). Secondly, EOC6 and EOC9 display a cobblestone morphology, whereas EOC8, EOC15, and EOC21 display a more spindle morphology (Supplementary Figure S3). It will be interesting to examine whether the more spindle morphology displayed by the recurrent EOC cells is associated with epithelial-mesenchymal transition (EMT) induced by chemotherapy. Finally, EOC8 isolated from a patient that was not responsive to treatment displayed the highest IC_50_ for carboplatin among the six primary EOC cells (Table 1 and Figure 3B). However, further studies involving a larger number of primary EOC samples are warranted to confirm these observations.

The most interesting finding in this study is that inhibition of p38 MAPK by SB203580 increases the number of viable cells in the primary cells in adherent culture or cultured in spheroids, which can be due to increased proliferation or decreased apoptosis. Because the neutral red uptake assay estimates the number of viable cells based on their ability to uptake the neutral red dye [41] cannot distinguish these two effects, further experiments are required to determine the effect of p38 MAPK activation on proliferation and apoptosis in the primary EOC cells. Inhibitor of apoptosis (IAP) proteins that include cIAP1 (cellular inhibitor of apoptosis protein-1), cIAP2, XIAP (X-linked inhibitor of apoptosis protein), and survivin promote survival of cancer cells by inhibiting apoptosis and have been proposed to be therapeutic targets in cancer [42,43]. In this regard, survivin has emerged as a potential therapeutic target for cancer treatment [43]. Recent studies showed that targeting survivin by short hairpin RNAs (shRNAs) or the inhibitor YM155 induces apoptosis in EOC cells and sensitizes EOC cells to cisplatin-induced apoptosis [34,44]. Furthermore, a recent study showed that inhibition of survivin expression by CRISPR/cas9 gene editing or YM155 decreased proliferation, migration, and invasion, as well as mesenchymal markers in two EOC cell lines [45]. These studies indicate that survivin is involved in multiple biological processes in EOC. Previous studies have shown that p38 MAPK is required for survivin downregulation in Tanshinone IIA and a high dose of nitric oxide-induced apoptosis in established EOC cell lines [21,35,36]. Our results show that inhibition of p38 MAPK by SB203580 increases survivin protein level in the primary EOC cells, which is consistent with the published results in established EOC cell lines [21,35,36]. However, further experiments are required to determine whether increased survivin contributes to SB203580-mediated increases in viable cells in the primary EOC cells. Our results showed that carboplatin abolishes survivin expression in primary EOC cells, even in the presence of SB203580, which may explain the inability of SB203580 to inhibit carboplatin-induced cell death in primary EOC cells.

In summary, our results show that pharmacological inhibition of p38 MAPK with SB203580 has minimal effect on carboplatin sensitivity in A2780s and primary EOC cells. Instead, it enhances carboplatin resistance in A2780cp cells and increases the number of viable cells in the primary EOC cells. Although further studies, especially in in vivo models, are necessary to further define the role of p38 MAPK in carboplatin sensitivity and proliferation/survival in EOC, our findings in this study suggest that pharmacological inhibition of p38 MAPK may not be a promising therapeutic strategy to treat EOC. Rather, the therapeutic potential of activating p38 MAPK could be explored. Currently there is an ongoing phase Ib/II clinical trial of p38 MAPK inhibitor LY2228820 for recurrent ovarian cancer to determine the effect of LY2228820 plus gemcitabine and carboplatin versus gemcitabine and carboplatin for women with platinum-sensitive ovarian cancer (https://clinicaltrials.gov/). The results of this clinical trial could provide useful information on the therapeutic value of targeting p38 MAPK in EOC.

## 4. Materials and Methods

### 4.1. Reagents

Neutral red dye, SB203580 (p38 MAPK inhibitor) and β-actin antibody (A5441) were purchased from Sigma-Aldrich (Oakville, Ontario, Canada). The protein inhibitor cocktail was purchased from Roche (Mississauga, Ontario, Canada). Antibodies against pan-keratin (#4545), p38 (#8690), phospho-p38 (#4511), cleaved PARP (#5625), PARP (#9542), p53 (#2527), phospho-p53 (Ser15) (#9284), phospho-p53 (Ser46) (#2521) phospho-p53 (Ser392) (#9281), survivin (#2808), phospho-MAPKAPK2 (#3007), MAPKAPK2 (#12155), phospho-Chk2 (#2179), Chk2 (#6334), and Alexa Fluor^®^ goat anti-rabbit secondary antibody (#8889) were purchased from Cell Signaling Technology (Danvers, MA, USA). The antibody against PAX8 (10336-1-AP) was purchased from Proteintech (Rosemont, IL, USA). 

### 4.2. Culture of Cell Lines

Human ovarian cancer cell lines A2780s and A2780cp cells were cultured in DMEM/F12 medium supplemented with 10% fetal bovine serum (FBS), 100 U/mL penicillin, and 100 µg/mL streptomycin. The cisplatin-resistant A2780cp cells were derived from cisplatin-sensitive A2780s cells by exposing A2780s cells to stepwise-increasing concentrations of cisplatin [46]. The paired A2780s (WT-p53) and A2780cp (p53 mutant, V172F and R260S) cells were provided by Dr. Benjamin Tsang (Ottawa Hospital Research Institute) [40].

### 4.3. Isolation and Culture of Primary EOC Cells

Primary EOC cells were isolated from ascites of high-grade serous EOC patients by following the protocol described by Shepherd et al. with minor modifications [47]. Briefly, we cultured a mixture of 50 mL ascites from EOC patients and 50 mL M199/MCDB105 medium supplemented with 10% fetal bovine serum (FBS), 100 U/mL penicillin, and 100 µg/mL streptomycin in T-125 flasks with 0.2 µm vented caps for three days prior to the first medium change. We then changed medium every three days until the cells became confluent. At this point, the hematopoietic cells were removed by medium change [47]. Cells with fibroblast contamination were discarded and excluded from the study. We then froze numerous vials of cells as passage 0 stocks in liquid N_2_. A portion of cells was re-seeded for experiments (passage 1). In this study, all EOC cells were used at passages 1–3. Institutional approval for research with human materials was received prior to the initiation of these studies (Health Research Ethics Board of Alberta-Cancer Committee, #25132), and samples were obtained after receiving informed consent. For spheroid culture, primary EOC cells were seeded into 24-well ultra-low attachment plates (Corning Incorporated, Corning, NY, USA) at a density of 50,000 cells per well in M199/MCDB105 medium supplemented with 10% fetal bovine serum (FBS), 100 U/mL penicillin, and 100 µg/mL streptomycin [48]. Clinicopathological information of the primary EOC cells are shown in Table 1. 

### 4.4. Immunocytochemistry

Immunocytochemistry was performed as previously described [49]. Briefly, cells growing on coverslips were fixed in 4% paraformaldehyde in phosphate-buffered saline for 10 min and permeabilized in 0.2% Triton X-100 for 10 min. The cells were incubated with affinity-purified rabbit anti-PAX8 antibody for 3 h (1:75 dilution), followed by Alexa Fluor^®^ (Carlsbad, CA, USA) goat anti-rabbit secondary antibody for 1 h (1:200 dilution) and 1 μg/mL DAPI (4′,6-Diamidino-2-Phenylindole, Dihydrochloride) for 5 min. Coverslips were mounted onto slides, and fluorescent images were captured using an AMG EVOS FL microscope (Mill Creek, WA, USA) with a 20× objective lens.

### 4.5. Proteome Profiler Human Phospho-Kinase Assay

A2780s and A2780cp cells were treated with 50 µM carboplatin for 24 h. We selected the dose of carboplatin and duration of the treatment based on our previous study [27]. We determined that the IC_50_ for carboplatin is 13.6 μM in cisplatin-sensitive A2780s cells and 150.8 μM in cisplatin-resistance A2780cp cells. At 50 μM, carboplatin killed approximately 80% of A2780s cells, but only about 15% of A2780cp cells at 72 h. More importantly, carboplatin at 50 μM did not induce any cell death at 24 h, allowing us to investigate the early signaling events that were involved in carboplatin-induced cytotoxicity. Cell lysates were collected and used for the phospho-kinase array experiments by following the manufacturer’s instructions (R&D, ARY003). The array data was quantified using NIH ImageJ (ImageJ1, NIH, Bethesda, MD, USA). 

### 4.6. Neutral Red Uptake Assay

The neutral red uptake assay estimates the number of viable cells based on their ability to uptake neutral red dye [41]. To determine carboplatin-induced cytotoxicity, we seeded cells into 96-well plates and treated them with increasing concentrations of carboplatin with or without co-treatment of 10 µM SB203580 for 72 h, and determined cell viability by the neutral red uptake assay as we previously described [27]. To determine the effect of SB203580 on cell growth, we cultured the primary EOC cells in adherent culture or in spheroid cultures (50,000 cells per well in 24-well ultra-low attachment plates) in the presence of 10 µM SB203580 or an equal volume of DMSO (vehicle control) for 48 or 72 h, and determined the cell numbers using the neutral red uptake assay as we previously described [27]. To measure the cell growth in the spheroids, we modified the neutral red uptake assay. Specifically, after the incubation with the neutral red for 3 h, the spheroids were pelleted and washed with PBS once prior to lysis in 110 µL lysis buffer (50% ethanol and 1% acetic acid). After lysis and brief centrifugation, 100 µL of the neutral red-containing supernatant was transferred into a 96-well plate for measurement of absorbance at 540 nm using a microplate reader.

### 4.7. Preparation of Whole Cell Lysates and Western Blotting

Whole cell lysates were prepared using modified radioimmunoprecipitation assay (RIPA) buffer as described previously [50]. Protein concentration was quantified using the DC™ (detergent compatible) protein assay (Bio-Rad, Mississauga, ON, Canada) and equal amount of proteins were loaded into each lane of a sodium dodecyl sulfate (SDS) polyacrylamide gel and transferred to nitrocellulose membrane. Immunoblotting was performed using the antibodies at 1:1000 dilution. Membranes were scanned and analyzed using an Odyssey^®^ IR scanner and Odyssey^®^ imaging software 3.0 (Li-COR, Lincoln, NE, USA). 

### 4.8. Statistical Analysis

Data are shown as mean ± SE of three to seven independent experiments. Statistical analysis and IC_50_ calculation was performed using GraphPad Prism 5 (GraphPad Software, La Jolla, CA, USA). Statistical significance between two groups was determined by paired *t*-test and defined as *p* < 0.05.

## Figures and Tables

**Table 1:** Clinicopathological information of the ascites-derived primary EOC cells

Sample ID	Age	Histological Type	Stage	Upfront Diagnosis/Recurrent	Response to Treatment
EOC6	75	High grade serous EOC	IV	Upfront diagnosis	Patient declined treatment, went to palliative care
EOC8	54	High grade serous EOC	IV	Rercurrent, previously treated with Carboplatin/Taxol and Gemcitabine	No response to first line chemotherapy, progressed on treatment
EOC9	46	High grade serous EOC	IIIC	Upfront diagnosis	Responsive to treatment, 15 months from treatment and no recurrence
EOC11	53	High grade serous EOC	IIIC	Recurrent, previously treated with Carboplatin/Taxol	Responsive to treatment, recurred at 12 months
EOC15	73	High grade serous EOC	IIIC	Recurrent, previously treated with Carboplatin/Taxol	Responsive to treatment, recurred at 7 months
EOC21	74	High grade serous EOC	IIIC	Recurrent, previously treated with Carboplatin	Responsive to treatment, recurred at 6 months

## Supplementary Materials

The following are available online at http://www.mdpi.com/1422-0067/19/8/2184/s1.
