# Supplementary material for: Pharmacological Inhibition of p38 MAPK by SB203580 Increases Resistance to Carboplatin in A2780cp Cells and Promotes Growth in Primary Ovarian Cancer Cells
Source: Int J Mol Sci. 2018 Jul 26;19(8):2184. doi: 10.3390/ijms19082184 (PMC6121386; doi:10.3390/ijms19082184)
Supplement: Supplementary file 1 [file ijms-19-02184-s001.pdf]

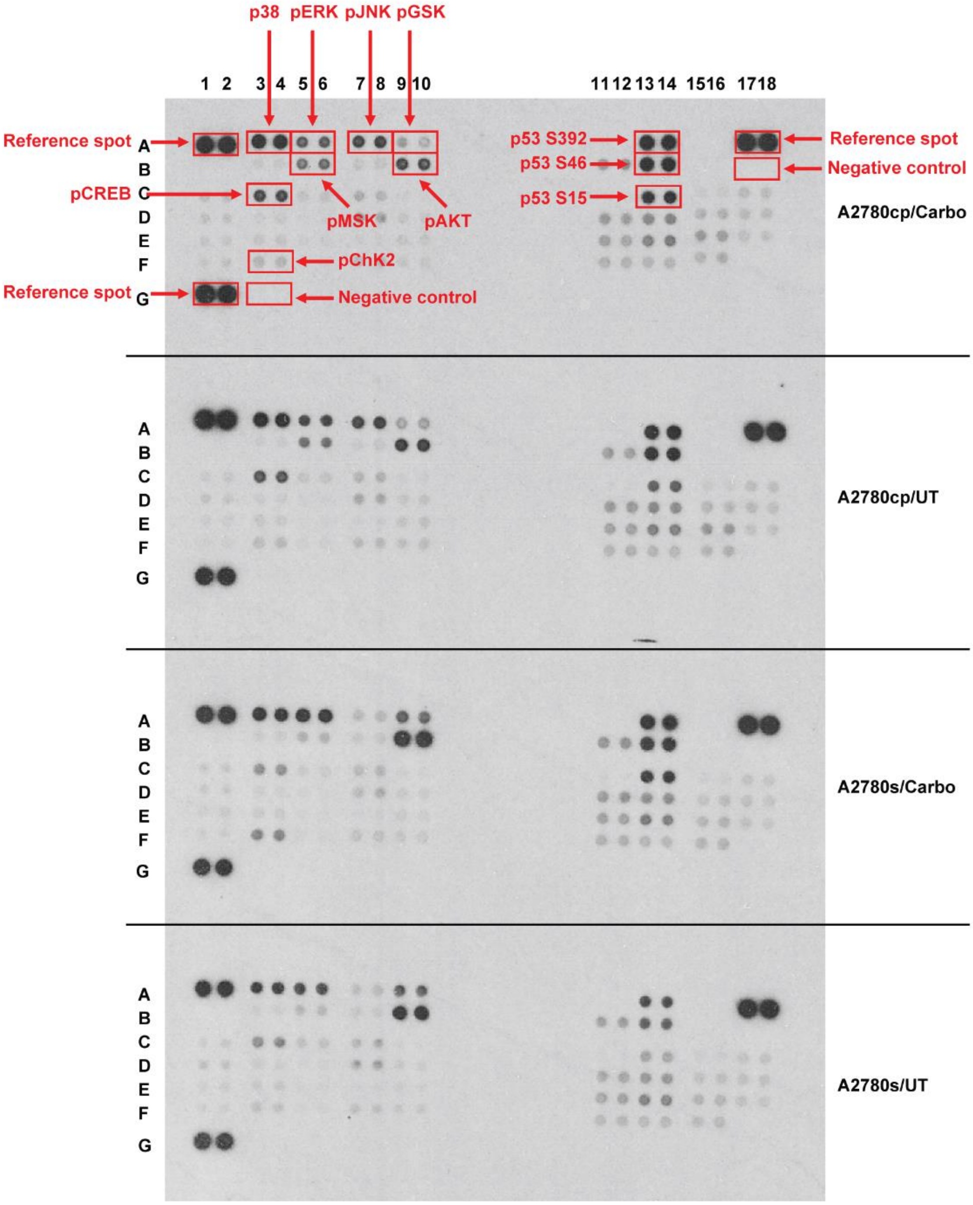

Supplementary Figure 1. A2780s and A2780cp cells display different phosphorylation profiles. A2780s and A2780cp cells were left untreated or treated with 50 μM carboplatin for 24 h. Cell lysates were collected and applied to the Human Phospho-Kinase Array analysis (R&D Systems, ARY033). Antibodies recognizing the phosphorylated signaling proteins or control antibodies were spotted in duplicate on the nitrocellulose membranes. The chemiluminescent signals of each spot was captured by exposure to X-ray films for various times (to avoid saturation of the signal). The X-ray image of one exposure time is shown. Spots of the differentially phosphorylated proteins, as well as the reference spots and negative control spots are marked.

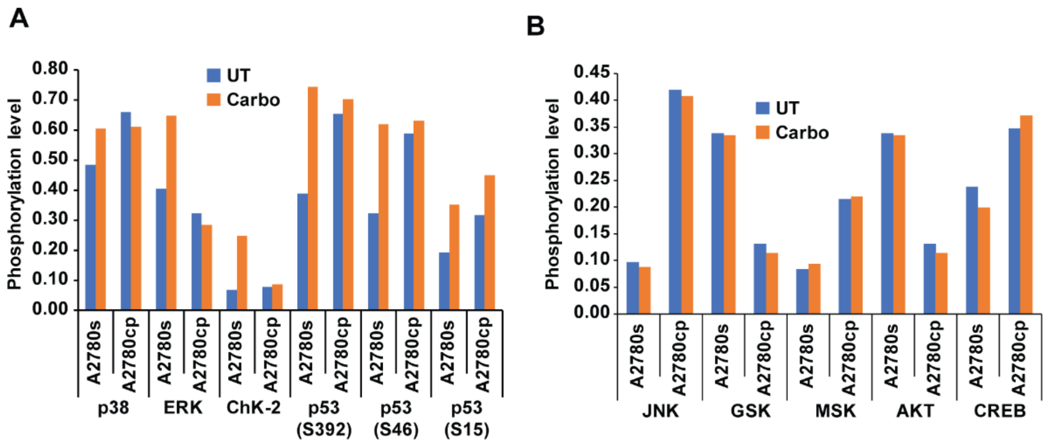

Supplementary Figure 2. Quantification of the phospho-kinase array data. The phospho-kinase array data was quantified using the NIH Image J and the differentially phosphorylated proteins are graphed. For quantification, the mean gray value of each spot was measured. Values of the same samples (two spots for each phospho-protein, 4 spots for the negative controls and 6 reference spots (positive controls) were averaged and used for calculation using the following formula: (mean gray value of a given phospho-protein - mean gray value of the negative control)/(mean gray value of the reference spot - mean gray value of the negative control). The phosphorylation level of each phospho-protein was expressed as ratio of the net mean gray value of the protein over that of the reference. The phosphorylation that was induced by carboplatin in A2780s cells is shown in **(A)** and the phosphorylation that was different between A2780s and A2780cp cells, but not induced by carboplatin is shown in **(B)**.

The phosphorylation sites and locations of the phospho-proteins on the arry (Supplementary Fig. 1) are as the following: p38 α (T180/Y182), A3 and A4; ERK1/2 (T202/Y204, T221/Y223), A5 and A6; JNK (T183/Y185, T221/Y223), A7 and A8; GSK α and β (S21/S9), A9 and A10; MSK1/2 (S376/S360), B5 and B6; AKT (S437), B9 and B10; CREB (S133), C3 and C4; ChK-2 (T68), F3 and F4; p53 (S392), A13 and A14; p53 (S46), B13 and B14; p53 (S15), C13 and C14.

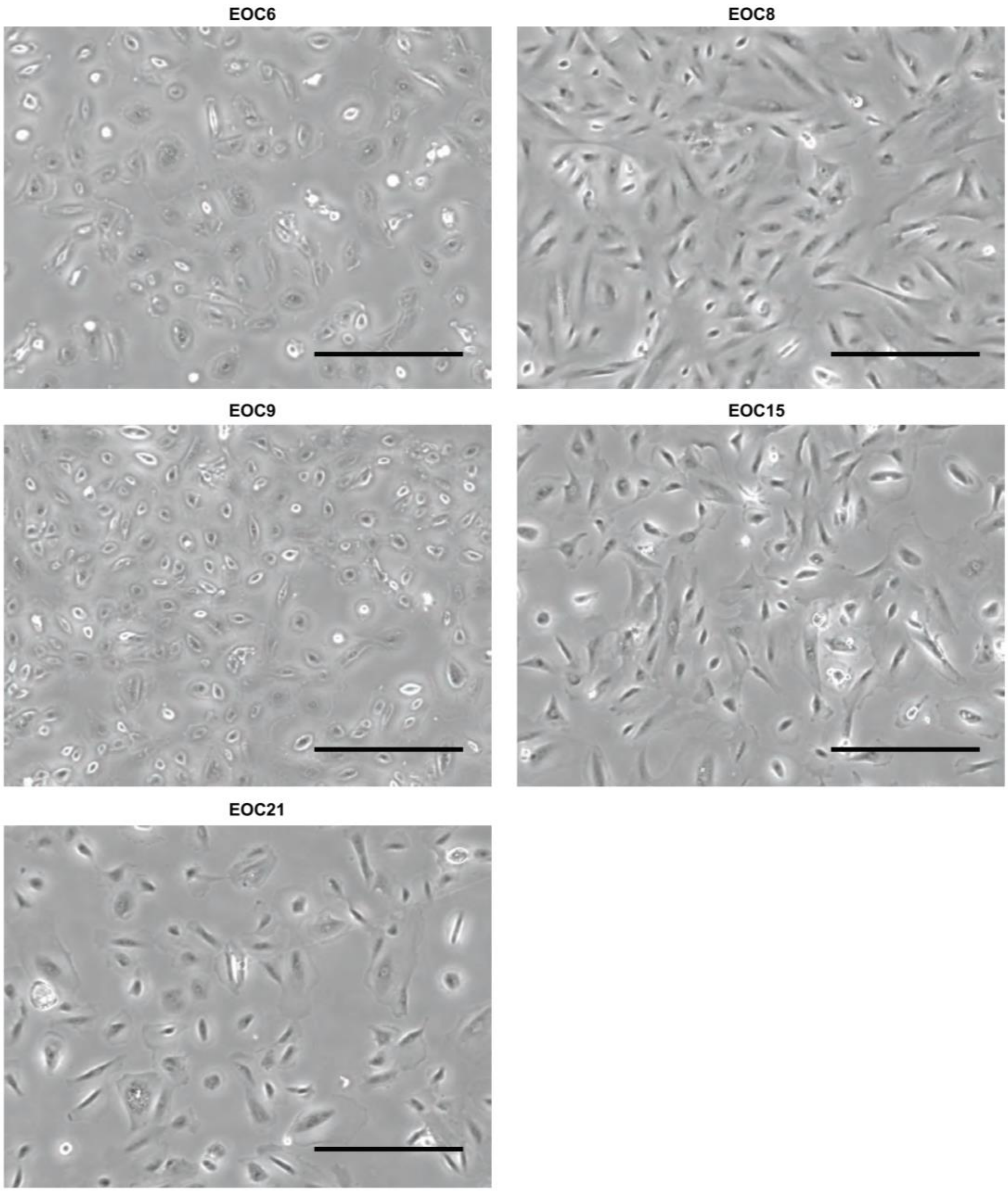

Supplementary Figure 3. Images of the ascites-derived primary EOC cells. Primary EOC cells were isolated from ascites of the high-grade serous EOC patients and cultured as described in the Material and Methods. Cell images were captured under the light microscope. The primary EOC cells display heterogeneity in cell morphology among the different patient samples. Scale bar = 250 μM.

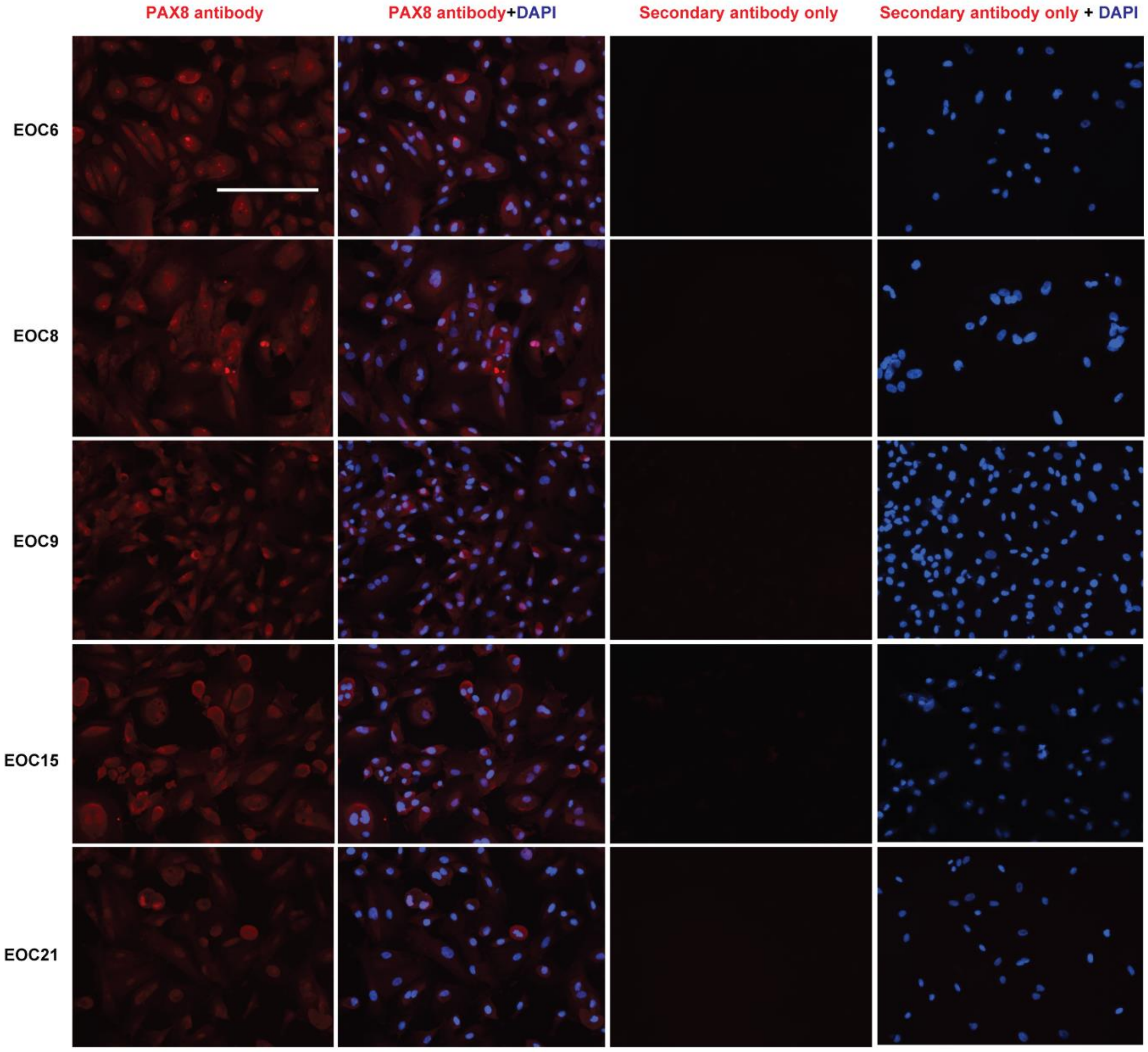

Supplementary Figure 4. Ascites-derived primary EOC cells express PAX8. Expression of PAX8 (a serous EOC marker) in the primary EOC cells was examined by immunocytochemistry suing an anti-PAX8 antibody. No antibody control (secondary antibody only) was used as the negative control. Nucleus was stained with DAPI. Scale bar = 200 μm.

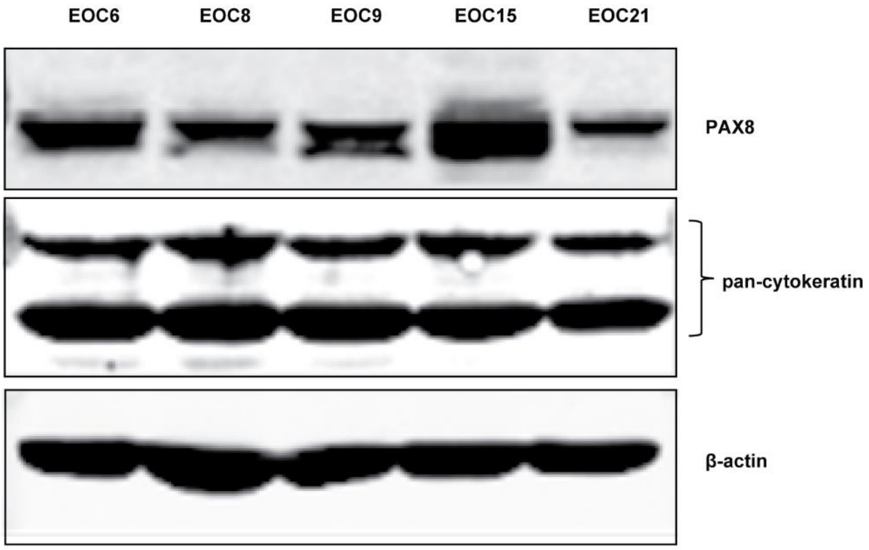

Supplementary Figure 5. Ascites-derived primary EOC cells express PAX8 and cytokeratins. Expression of PAX8 (a serous EOC marker) and cytokeratins (epithelial cell marker) in the primary EOC cells was examined by Western blotting using an anti-PAX8 and anti-pan-keratin antibody, respectively. β-actin was used as the loading control.

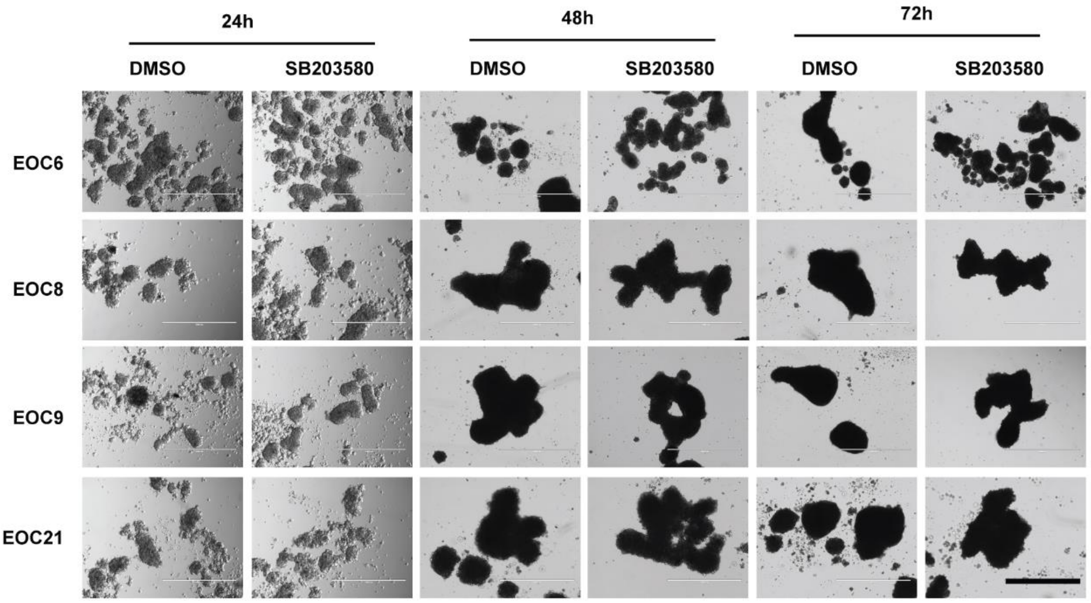

Supplementary Figure 6. Images of the spheroids formed by primary EOC cells. Primary EOC cells were seeded into 24-well ultra-low attachment plates in the presence of 10 μM SB203580 or an equal volume of DMSO. Images of the spheroids were captured under a light microscope at 24h, 48h and 72h. Scale bar = 1000 μm. A more visible scale bar was added manually to the last image (EOC21/SB203580/72h) to help estimate the size of the spheroids. Primary EOC cells readily formed spheroids after the 24 h. After that, the spheroids aggregated to form larger and more compact clusters as shown at the 48 and 72 h.
